# Deciphering transcript architectural complexity in bacteria and archaea

**DOI:** 10.1128/mbio.02359-24

**Published:** 2024-09-17

**Authors:** John S. A. Mattick, Robin E. Bromley, Kaylee J. Watson, Ricky S. Adkins, Christopher I. Holt, Jarrett F. Lebov, Benjamin C. Sparklin, Tyonna S. Tyson, David A. Rasko, Julie C. Dunning Hotopp

**Affiliations:** 1 Institute for Genome Sciences, University of Maryland School of Medicine, Baltimore, Maryland, USA; 2 Department of Microbiology and Immunology, University of Maryland School of Medicine, Baltimore, Maryland, USA; 3 Center for Pathogen Research, University of Maryland School of Medicine, Baltimore, Maryland, USA; 4 Department of Microbial Pathogenesis, University of Maryland School of Dentistry, Baltimore, Maryland, USA; 5 Greenebaum Cancer Center, University of Maryland School of Medicine, Baltimore, Maryland, USA; University of Cambridge, Cambridge, United Kingdom

**Keywords:** transcriptomics, bacterial transcripts, archaeal transcripts, small RNAs, non-coding RNA (ncRNA), direct RNA sequencing

## Abstract

RNA transcripts are potential therapeutic targets, yet bacterial transcripts have uncharacterized biodiversity. We developed an algorithm for transcript prediction called tp.py using it to predict transcripts (mRNA and other RNAs) in *Escherichia coli* K12 and E2348/69 strains (Bacteria:gamma-Proteobacteria), *Listeria monocytogenes* strains Scott A and RO15 (Bacteria:Firmicute), *Pseudomonas aeruginosa* strains SG17M and NN2 strains (Bacteria:gamma-Proteobacteria), and *Haloferax volcanii* (Archaea:Halobacteria). From >5 million *E. coli* K12 and >3 million *E. coli* E2348/69 newly generated Oxford Nanopore Technologies direct RNA sequencing reads, 2,487 K12 mRNAs and 1,844 E2348/69 mRNAs were predicted, with the K12 mRNAs containing more than half of the predicted *E. coli* K12 proteins. While the number of predicted transcripts varied by strain based on the amount of sequence data used, across all strains examined, the predicted average size of the mRNAs was 1.6–1.7 kbp, while the median size of the 5′- and 3′-untranslated regions (UTRs) were 30–90 bp. Given the lack of bacterial and archaeal transcript annotation, most predictions were of novel transcripts, but we also predicted many previously characterized mRNAs and ncRNAs, including post-transcriptionally generated transcripts and small RNAs associated with pathogenesis in the *E. coli* E2348/69 *LEE* pathogenicity islands. We predicted small transcripts in the 100–200 bp range as well as >10 kbp transcripts for all strains, with the longest transcript for two of the seven strains being the *nuo* operon transcript, and for another two strains it was a phage/prophage transcript. This quick, easy, and reproducible method will facilitate the presentation of transcripts, and UTR predictions alongside coding sequences and protein predictions in bacterial genome annotation as important resources for the research community.

**IMPORTANCE:**

Our understanding of bacterial and archaeal genes and genomes is largely focused on proteins since there have only been limited efforts to describe bacterial/archaeal RNA diversity. This contrasts with studies on the human genome, where transcripts were sequenced prior to the release of the human genome over two decades ago. We developed software for the quick, easy, and reproducible prediction of bacterial and archaeal transcripts from Oxford Nanopore Technologies direct RNA sequencing data. These predictions are urgently needed for more accurate studies examining bacterial/archaeal gene regulation, including regulation of virulence factors, and for the development of novel RNA-based therapeutics and diagnostics to combat bacterial pathogens, like those with extreme antimicrobial resistance.

## INTRODUCTION

**Fig 1:**
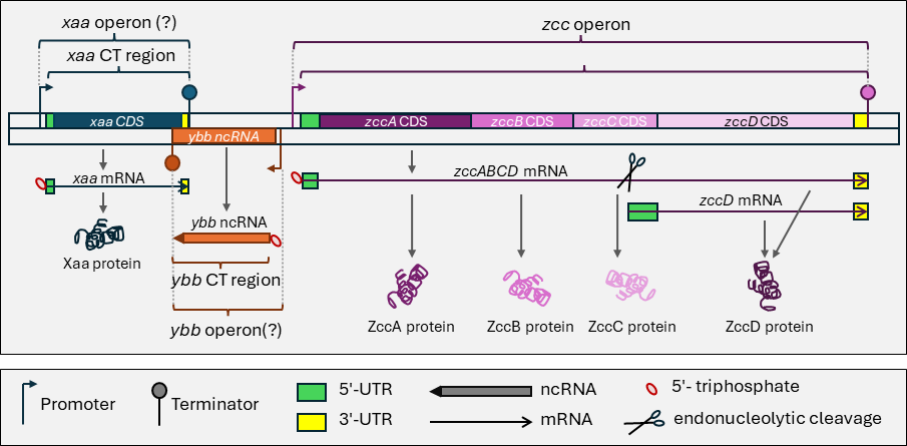
Overview of transcript, operon, and UTR definitions used. The interrelationship of genomic features described in this manuscript is illustrated, including the relationship of operon, CT region, CDS, mRNA, ncRNA, and proteins for monocistronic/polycistronic transcripts with/without transcript isoforms. The genes and genome are fictitious and used merely to illustrate the definitions of key terms.

Genomics, genome-enabled technologies, computational biology, and large-scale data mining are essential for rigorous, modern experiments on all organisms. Whole-genome sequencing and protein-based annotation are now routine, low-cost approaches for analyzing bacteria and archaea. But often the annotation, and thus analysis and experimental validation, is limited to predicted protein-coding regions and a few highly conserved non-coding RNAs (ncRNAs) like the rRNAs and tRNAs. Yet, pathogen RNA transcripts, particularly ncRNAs and RNA-mediated regulation, offer an unexplored set of druggable targets, diagnostics, and potential therapeutics (1). In this context, a transcript is a physical RNA molecule made from a DNA template that has discrete start and end sites generated by a diversity of molecular mechanisms (e.g., promoter/terminator and post-transcriptional processing) (Fig. 1).

In bacteria, transcripts are frequently considered within the paradigm of operons as put forth by Jacob and Monod (2), which was summarized recently as “sets of contiguous and functionally related genes cotranscribed from a single promoter up to a single terminator” (3), including the operator regulatory region (Fig. 1). Using this definition, polycistronic transcripts are encoded within operons, which also include regulatory regions. It is unclear if a monocistronic transcript and its regulatory regions would also be considered an operon. Operons are widespread in bacterial/archaeal genomes, with ~630–700 defined operons in *Escherichia coli* (4). Experimentalists have predicted operons using read counts and/or sequencing depth without algorithms [e.g., references (5, 6)], and efforts have been made to develop algorithms for their prediction (7–13). For example, the Rockhopper algorithm predicts operons using a naïve Bayes classifier to combine strand, intergenic distance, and coordinated differential expression in a unified probabilistic model (14).

Oftentimes, bacterial transcripts and operons are conflated, but fundamentally, the classical definition of operon is a DNA-based definition, defining a region in DNA that extends beyond the RNA-based transcripts to include the promoter/operator and terminator. Operons can have multiple transcripts due to post-transcriptional processing (15), alternate terminators (e.g., attenuation) (10, 16, 17), and alternate transcriptional initiation sites (3). There is a need for both DNA-based annotation of operons and RNA-based annotation of transcripts. Fundamentally, RNA-seq is transcript quantification; therefore, it should be measured at the RNA/transcript level not the DNA/operon level. Rockhopper has been used for differential expression of its predicted operons (11), but it yields different results than a corresponding transcript-focused analysis (3).

Fundamental biological differences such as a high coding density and polycistronic transcripts in bacterial genetics mean that we cannot merely apply the same laboratory and computational methods that were designed and optimized for humans and eukaryotic model organisms, with the false assumption that they will work because bacteria are “simpler” than humans. Currently most bacterial/archaeal RNA-seq studies are conducted by applying tools designed for eukaryotic transcripts using bacterial coding sequence (CDS) predictions. Even when issues with counting algorithms are mitigated for a CDS-focused analysis of polycistronic transcripts (18), measurements of CDSs in polycistronic transcripts are dependent on one another yet are treated as independent measurements with the statistics used to detect differential expression. This results in errors in variance estimations in differential expression tools (19). Comparisons of the StringTie algorithm for transcript prediction and Rockhopper have previously noted some of these issues, as well as the need for long RNA sequence reads to resolve these problems (10).

*E. coli* K12 is a well-studied genome that has some transcript predictions (17, 20), anti-sense RNA characterization (21), and transcriptional start site and terminator predictions (17, 22–25), all of which are aggregated and manually curated in RegulonDB (26) and EcoCyc (27). But even for this well-studied organism, reference annotation files (like GFF or GTF files) lack transcript annotations, and it can be difficult, if not impossible, to ascertain and use transcript structures for a differential expression analysis. The current work done to characterize transcripts and transcriptional regulation in *E. coli* [e.g., reference (26)] is not possible for more than a few microorganisms, yet there is immense bacterial biodiversity. Therefore, we sought to develop a fast, simple, rigorous, and reproducible method for identifying bacterial transcripts that can be widely applied and takes advantage of recent advances in RNA sequencing, including PacBio IsoSeq and Oxford Nanopore Technologies (ONT) direct RNA sequencing both of which have been applied previously to bacteria including *E. coli* (3, 28–30). Transcript predictions will enable differential expression analyses to be expanded to include ncRNAs and also use the latest transcript-based differential expression analysis tools like Salmon (31) and Kallisto (32). Transcript predictions are also needed to inform the consequences of genetic knock-in and knock-out experiments [e.g., reference (33)], identify regulatory sequences [e.g., references (10, 16, 34)],and detect post-transcriptional processing [e.g., references (15, 35)]. Recent studies (10, 28, 36) reveal a much more complex picture of bacterial transcripts with post-transcriptional processing and potentially multiple promoters and terminators, including transcripts beginning or ending in the middle of adjacent coding sequences due to the coding density (17).

In this study, we describe a quick, easy, and reproducible method and algorithm for whole transcriptome sequencing and structural annotation using ONT direct RNA sequencing. We tested the methods on the *E. coli* K12 and E2348/69 strains and then also applied this algorithm to existing public data for *Pseudomonas aeruginosa* strains SG17M and NN2 (37), *Listeria monocytogenes* strains Scott A and RO15 (38), and *Haloferax volcanii* (39).

## RESULTS

### ONT direct RNA sequencing of *E. coli* transcripts

**Fig 2:**
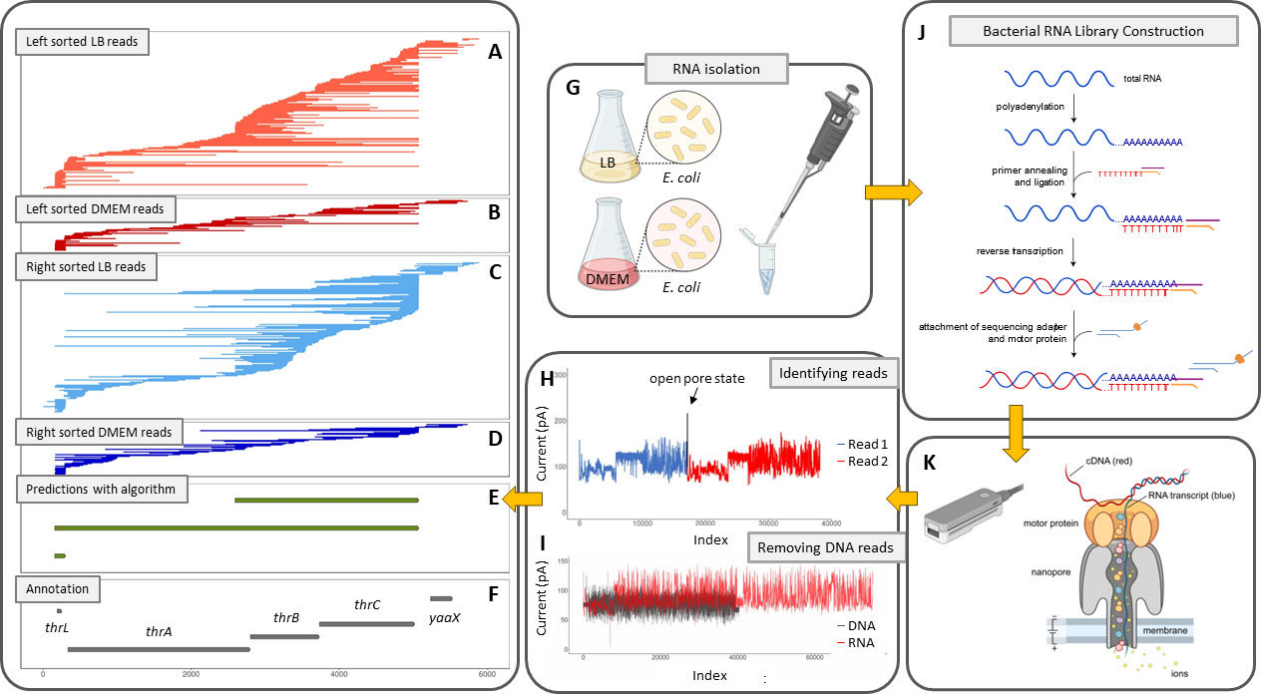
Overview of the experimental/analysis workflow. Plus-strand ONT direct RNA sequencing reads (shown as lines) are mapped from 1 bp to 6 kbp in the *E. coli* K12 genome (NC_000913.3), which corresponds to the *thr* operon, and sorted by their transcription stop site for *E. coli* K12 grown in rich LB media [left sorted (A); right sorted (**C**)] and DMEM media [left sorted (B); right sorted (**D**)]. Our algorithm predicts three transcripts (**E**), and four CDSs in the annotation file are illustrated (**F**). The transcript for the leader peptide *thrL* is recovered in both growth conditions. (**G**) RNA was isolated from *E. coli* K12 grown at 37°C with aeration in LB and DMEM media. (**H**) Squiggle plot for two sequencing reads in tandem. In this case, the open pore state was missed by the software resulting in a chimeric read. In both reads, the DNA adapter can be observed with lower current followed by a relatively flat plateau that corresponds to the polyA tail. This is followed by the electrical current changes associated with the RNA moving through the pore. (**I**) Plots show the electrical current for the same length DNA and RNA highlighting that the signal-to-base ratio is different for RNA and DNA. (**J**) The standard ONT direct RNA sequencing library was used on bacterial RNA that was *in vitro* polyadenylated following RNA isolation. Library construction and (**K**) loaded on an ONT MinION device for nanopore sequencing.

We generated ONT direct RNA sequencing data (Fig. 2) from RNA isolated from *E. coli* K12 and pathogenic *E. coli* E2348/69 (40) grown at 37°C with aeration in LB and DMEM media (Table 1; Table S1), which are virulence gene inducing growth conditions (15, 41–44). *E. coli* K12 annotation is available for comparison in RegulonDB (26) and EcoCyc (27) and includes transcript predictions (17, 20), anti-sense RNA characterization (21), and transcriptional start site and terminator predictions (17, 22–25). The inclusion of *E. coli* E2348/69 allows us to interrogate transcript predictions in a related but clinically relevant enteropathogenic *E. coli* (EPEC) strain with plasmids (40) that have pathogenesis-associated operons, which have had fine-scale analysis of transcription (15, 44). We focused on using ONT direct RNA sequencing, where RNA was sequenced directly in the pore (Fig. 2K), to predict bacterial transcripts (Fig. 2E) because it does not have template switching (36). Additionally, ONT direct RNA sequencing data lack genomic DNA contamination since sequenced RNA and DNA have markedly different signals, which is used by Guppy to eliminate DNA reads with high fidelity. RNA advances through the pore more slowly and with a higher electrical current range than DNA, which is apparent in all RNA reads since RNA is loaded into the pore using a ligated DNA adaptor (Fig. 2I; Fig. S1).

**TABLE 1:** Characteristics of predicted transcripts for *Escherichia coli, Listeria monocytogenes*, and *Pseudomonas aeruginosa*

Feature	*Escherichia coli* K12 (GCF_000005845.2)	*Escherichia coli* E2348/69 (GCF_014117345.2)	*Listeria monocytogenes* Scott A (CM001159.1)	*Listeria monocytogenes* RO15 (CADEHJ000000000.1)	*Pseudomonas aeruginosa* SG17M (NZ_CP080369.1)	*Pseudomonas aeruginosa* NN2 (NZ_LT883143.1)	*Haloferax volcanii* (GCF_000025685.1)
Number of contigs in reference	1	3	1	2	1	1	5
Number of reads used	5,266,309	3,025,047	1,679,073	1,664,744	220,553	1,196,279	1,438,670
Number of CT regions for predictions (>20 reads)	1,055	1,071	525	464	391	1,209	640
Number of regions on (+)-strand	521	528	238	206	181	612	318
Number of regions on the (−)-strand	534	543	287	258	210	597	322
Span (bp) on (+)-strand	2,068,709	1,951,551	703,660	589,005	530,329	1,944,294	893,429
Span (bp) on (−)-strand	2,135,707	1,827,581	821,637	759,698	589,348	1,886,100	974,115
Average span (bp) +strand	3,968	3,777	2,946	2,848	2,915	3,174	2,807
Average span (bp) – strand	3,997	3,446	2,851	2,932	2,786	3,155	3,022
Number of transcripts	3,618	2,248	881	793	274	1,103	613
Number of transcripts on the (+)-strand	1,465	1,101	402	361	79	495	241
Number of transcripts on the (−) strand	2,153	1,147	479	432	195	608	372
Number of regions with one transcript	289	429	218	199	85	258	226
Maximum number of transcripts per region	254	141	32	31	68	63	27
Mean 3′-UTR (bp)	150	126	122	112	163	236	180
Median 3′-UTR (bp)	72	62	48	47	59	78	84
Maximum 3′-UTR (bp)	2,716	1,261	1,306	1,245	2,235	2,809	2,040
Mean 5′-UTR (bp)	134	119	137	114	185	205	373
Median 5′-UTR (bp)	53	49	36	33	93	85	207^*a*^
Maximum 5′-UTR (bp)	2,122	2,817	2,303	2,303	1,835	1,943	2,955
Number of genes	4,494	4,809	3,038	3,149	6,349	6,380	3,956
Number of genes in annotated transcript	2,360	2,037	765	680	209	765	385
Number of genes associated with just one transcript	1,341	1,300	636	554	168	572	301
Maximum number of transcripts a single gene is associated with	15	12	6	7	4	6	10
90% of genes are associated with fewer than this number transcripts	4	4	3	3	3	3	3
Number of transcripts with one gene	1,563	1,096	349	316	79	398	167
Maximum number of genes in a single mRNA	17	14	38	22	15	15	15
90% of transcripts have fewer than this many genes	4	4	4	3	3	3	3
Number of predicted mRNAs	2,487	1,844	536	491	133	601	263
Average predicted mRNA size (bp)	1,617	1,732	1,660	1,607	1,590	1,735	1,948
Largest predicted mRNA (bp)	13,305	15,256	29,034	10,791	14,168	12,709	10,463
Smallest predicted mRNA (bp)	131	129	224	209	183	146	136
Number of predicted ncRNAs (including ones in reference annotation file)	1,131	404	345	302	141	502	350^*a*^
Average predicted ncRNA size (bp)	550	649	497	524	578	538	724^*a*^
Largest predicted ncRNA (bp)	2,947	2,916	2,585	2,588	6,361	2,851	3,045^*a*^
Smallest predicted ncRNA (bp)	89	80	95	136	97	77	81^*a*^
Genes in longest mRNA	g*lf, gnd, insH7, rfbABCDX, wbbHIJKL*	*nuoABCEFGHIJKLMN*	phage (LMOSA_9400-LMOSA_9770)	*rplBCDEFNOPRVWX, rpmCD, rpsCEHJQS, secY*	*fusA,rplJL,rpoBC,rpsGL, tuf*	phage (PANN_06920 - PANN_07050)	*nuoABCD1HIJ1J2KLMN*

^*a*^ The reads for this species frequently do not extend beyond the 5′-end of the CDS, essentially meaning transcripts start where translation is predicted to start. When this happens for a polycistronic transcript, the result is a very long 5′-UTR as seen with the increased median, and when this happens for a monocistronic transcript, the mRNA is erroneously called a ncRNA. While this likely occurs for all of the organisms, it is acute for the *H. volcanii* data. It may be that the 5′-end predictions of the CDS are flawed due to calling the longest ORF, or it may be that the *H. volcanni* UTRs are shorter than the bacterial 5′-UTRS.

### Predicted *E. coli* K12 transcripts

Using the 5,266,309 ONT reads generated for *E. coli* K12 (Table 1), we predicted transcripts using the algorithm that we developed to predict transcripts in prokaryotic genomes using ONT sequencing reads first predicting transcript start/stop sites where there is an overabundance of reads starting/ending and then identifying start/stop site combinations supported by the ONT sequencing data using models based on the observed characteristics of ONT sequencing, which is described in more detail below. We identified 3,902 strand-specific contiguously transcribed (CT) regions in the K12 genome with 1,055 that had >20 reads that we used for predictions (Table 1). The 1,055 CT regions used for predictions were on average 4 kbp and included 521 regions on the (+)-strand spanning 2.07 Mbp and 534 regions on the (–)-strand spanning 2.14 Mbp (Table 1). There were 3,618 predicted transcripts with 1,465 predicted transcripts on the (+)-strand and 2,153 predicted transcripts on the (–)-strand (Table 1). There were 289 (27%) regions with only a single transcript predicted (Table 1), meaning 73% of CT regions contained more than one transcript either because operons overlap or because there were multiple overlapping transcripts in an operon.

Of the 3,618 predicted transcripts, 2,484 were predicted to be mRNAs (Fig. 1) and 1,134 were predicted to be ncRNAs (Fig. 1; Table 1). mRNAs were defined as transcripts that have at least one annotated CDS found completely within the transcript boundaries, whereas a ncRNA was defined as a transcript that lacks a CDS found completely within the transcript boundaries (Fig. 1). It is important to note that frequently the 5′-end of CDSs (and the N-terminal portion of the protein encoded by them) are incorrectly annotated, such that the assignment of transcripts as mRNA/ncRNA needs further manual refinement including possible curation of the N-termini of proteins; additionally, protein annotation may be informed and improved through transcript structural annotation. However, given these definitions, the average mRNA was 1,618 bp with the smallest and largest being 131 and 13,305 bp, respectively (Table 1). The average ncRNA was 517 bp with the smallest and largest being 52 and 2,947 bp, respectively (Table 1). Of these 1,134 predicted ncRNAs, 23 (2%) were already described in the reference annotation file and are ~23% of the 98 previously annotated ncRNAs in the reference annotation file (Table 1).

**Fig 3:**
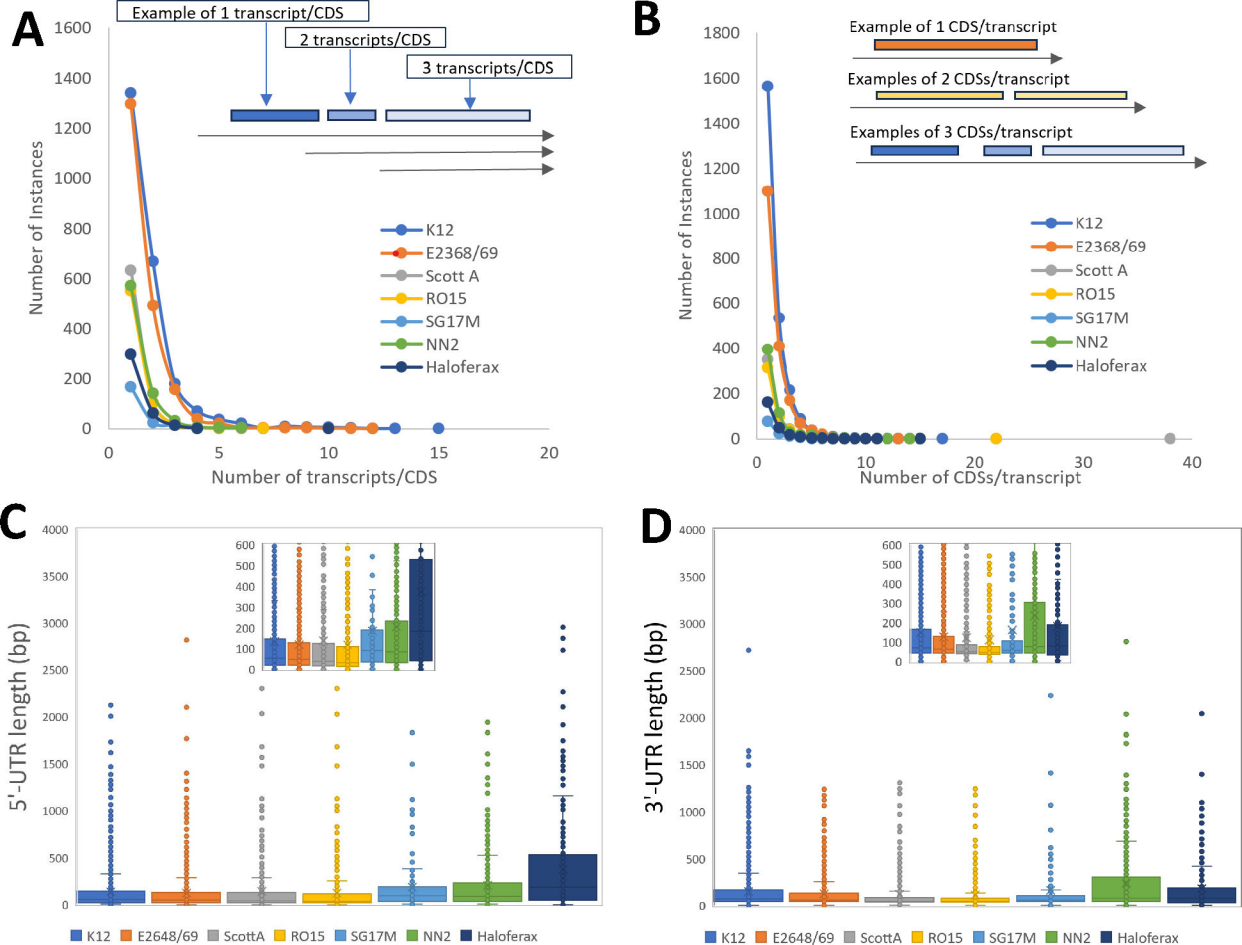
Characteristics of transcript predictions. The distribution of the number of instances of CDS by transcripts/CDS (**A**) and the distribution of the number of instances of transcripts by CDSs/transcript (**B**) are shown for *E. coli* K12, *E. coli* E2368/69, *L. monocytogenes* ScottA, *L. monocytogenes* RO15, *P. aeruginosa* SG17M, *P. aeruginosa* NN2, and *H. volcanii*. The data points in these discrete distributions are connected by lines for visualization purposes. The inset in each illustrates how transcripts/CDS and CDSs/transcript are defined. The size distributions of predicted 5′-UTRs (**C**) and 3′-UTRs (**D**) are plotted for each of the six strains examined with an inset that zooms in on 0–350 bp to better illustrate the distribution of the majority of the data.

Of the 4,494 annotated CDSs, 2,357 were in an annotated transcript while 2,775 were not, suggesting that with these growth conditions we annotated transcripts associated with half of the predicted CDSs, which is consistent with previous results (45). Of those, 1,341 (57%) CDSs were associated with a single transcript and 90% of CDSs were associated with <4 transcripts (Table 1; Fig. 3A). While 1,564 of the predicted transcripts contained only a single CDS (Table 1; Fig. 3B), the predicted transcript with the largest number of CDSs encoded within it contained 17 CDSs, including *glf*, *gnd*, *insH7*, *rfbABCDX*, and *wbbHIJKL* (Table 1).

Using the predicted mRNAs (excluding ncRNAs) and CDSs, we predicted the 5′- and 3′-untranslated regions (UTRs). The median 5′-UTR was 53 bp and the most common length (mode) was 14 bp, while the median 3′-UTR was 72 bp, and the most common length (mode) was 36 bp (Table 1; Fig. 3C and D). This is consistent with previous reports that the 5′-UTR is 20–40 nt (24), despite previous reports that ONT sequencing cannot capture the terminal 5′-end of transcripts (39).

### Complexity of bacterial transcription

Our predictions detect tremendous bacterial transcript structural variation while confirming previous experimentally verified predictions. For example, in the *thr* operon, three transcripts were predicted, including the previously described *thrL* transcript for the leader peptide, the *thrLABC* transcript, and a *thrBC* transcript (46) (Fig. 2E).

**Fig 4:**
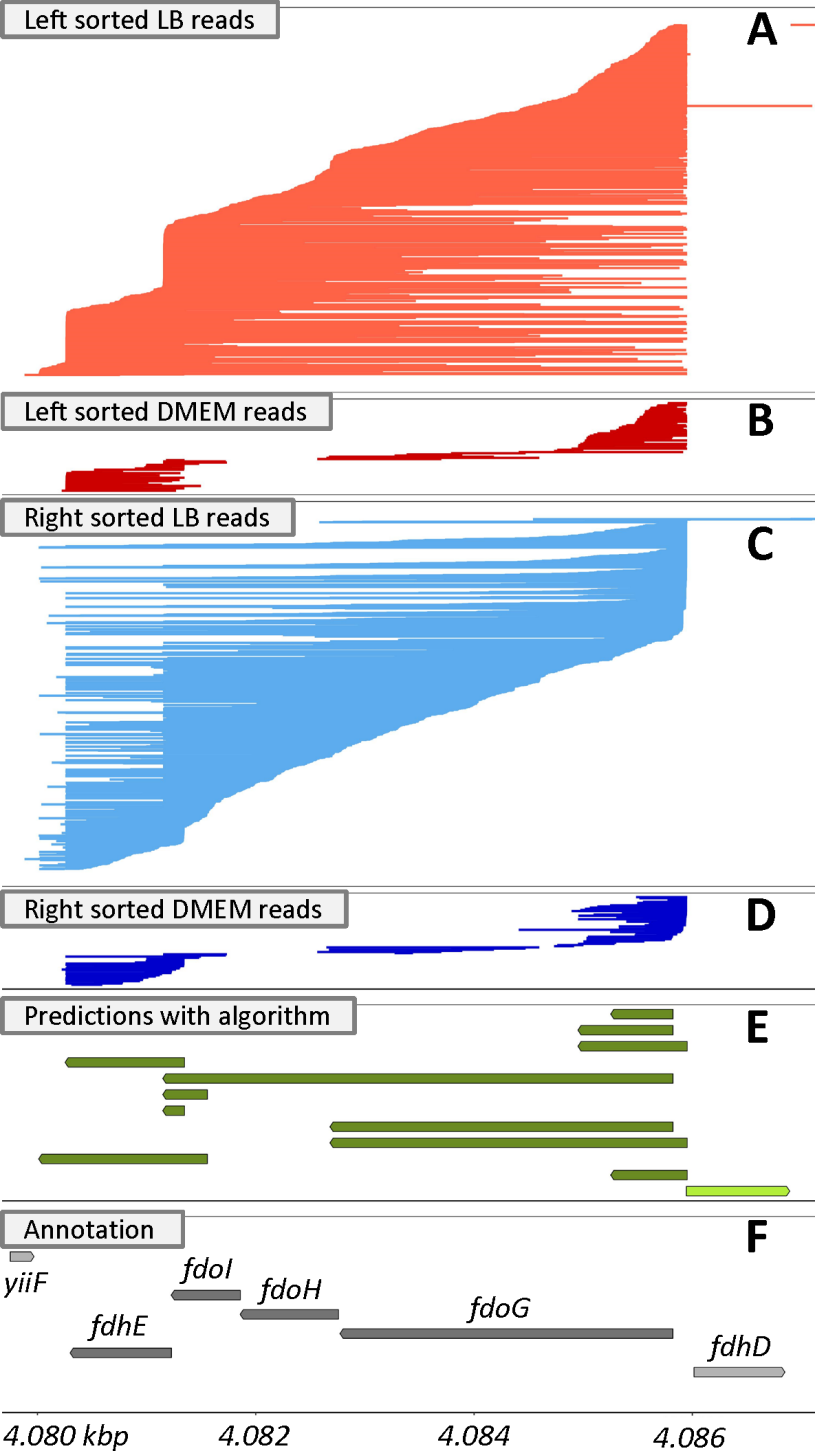
fdoGHI-fdhE Transcripts. Reads mapping to the minus strand of the *E. coli* K12 genome (NC_000913.3) grown in LB (**A, C**) and DMEM (**B, D**) are shown for a region from 4,080-4,088 kbp. To facilitate the visualization of the starts and stops of transcripts, reads were sorted by either their left most (**A, B**) or right most (**C, D**) position and plotted from top to bottom accordingly. Transcript predictions from our algorithm (**E**) and the predicted CDSs in the reference annotation file (**F**) are shown with arrows indicating the direction of transcription and with transcripts/CDSs on the different strands having different shading (light for the (+)-strand and dark for the (-)-strand).

Other regions were more complex, like the region from 4,080 to 4,087 kbp encompassing *fdoGHI* and *fdhE* (Fig. 4). RegulonDB (26) and EcoCyc (27) describe this entire region as an operon with two promoters—one that makes a transcript for the entire region and a second smaller internal transcript encoding *fdhE* that is started from a promoter within *fdoH* (Fig. 4). The ONT data suggested differential expression of the transcript isoforms where *fdoGHI* was largely untranscribed in DMEM relative to LB while *fdhE* was transcribed in both (Fig. 4). A small ncRNA was observed in DMEM when *fdoG* was not transcribed. (Fig. 4). We predicted 11 different transcripts in this entire region, including the *fdhE* transcript that started in *fdoH* (Fig. 4). This algorithm likely underpredicted long transcripts, due to the limitations of the ONT technology as described below. So despite evidence for a complete *fdoGHI-fdhE* transcript, we did not predict it, likely because there was insufficient sequencing depth (Fig. 4). But there was robust evidence for many of the other transcripts predicted that were not currently in RegulonDB, EcoCyc, or the annotation file, including a transcript of just *fdoG*, just *fdoGHI*, two putative overlapping small RNAs that overlap the end of *fdoI* and the beginning of the *fdhE* transcript, and four putative overlapping small RNAs that overlap the beginning of *fdoG* (Fig. 4). In a typical differential expression analysis that uses CDS regions, these four putative small RNAs overlapping *fdoG* would likely be misinterpreted as expression of *fdoG* in DMEM. Importantly, while we detected these transcripts, we cannot ascertain that they have a function, and they could merely be stable degradation products of transcription. Regardless, they are likely to confound and obfuscate differential expression analyses.

Across the 11 transcripts predicted in the *fdoGHI/fdhE* region, there was variation in transcript start and end sites, as previously described (15, 24). This variability included slightly longer transcripts that extend beyond *fdhE* that are observed under both growth conditions and were reproducible across all sequencing runs (Fig. 4). This variability was seen in many regions, suggesting that transcription initiation and termination are flexible.

### Predicted *E. coli* E2348/69 transcripts

The 60% fewer reads sequenced for *E. coli* E2348/69 relative to K12 led to fewer transcript predictions (Table 1), particularly fewer ncRNA predictions, but otherwise the results are quite similar. The longest predicted mRNA for E2348/69 was *nuoABCEFGHIJKLMN*, a known operon (47, 48). Unlike the K12 strain, the E2348/69 strain contains two plasmids (NZ_CP059841.1 and NZ_CP059842.2, respectively) and mRNA and ncRNAs were predicted on both plasmids. Of the four ncRNAs in the reference annotation, we predicted two (*rnpB* and *ssrS*). Additional known ncRNAs missing in the reference annotation file were identified, including *glmY* and *glmZ*, both of which are important for the regulation of the *LEE* operon and virulence (44).

**Fig 5:**
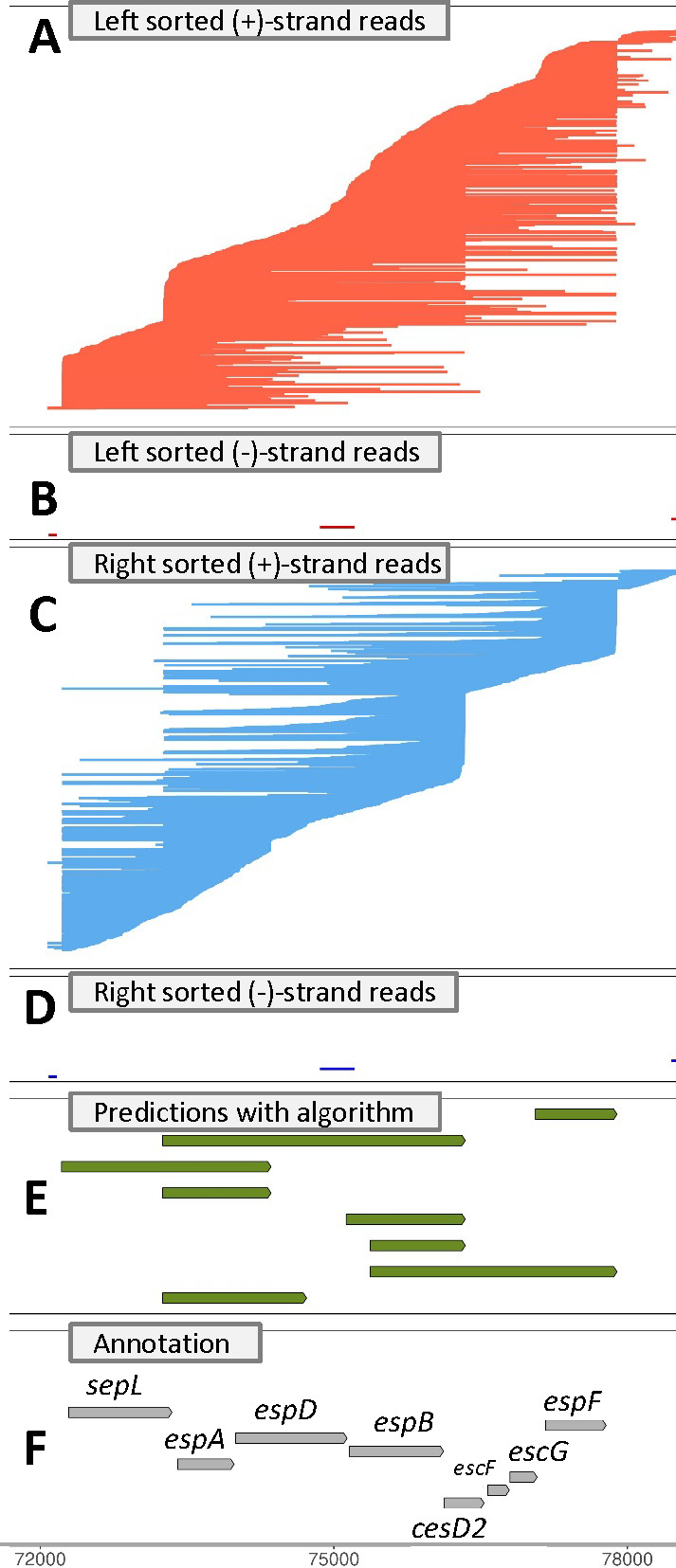
LEE4 operon. Reads are illustrated that map to the plus strand (**A, C**) and minus strand (**B, D**) of the *E. coli* E2348/69 genome (GCF_014117345.2) grown in LB or DMEM for a region from 72 to 78 kbp. There are no reads from the LB conditions on the (+)-strands. To facilitate the visualization of the starts and stops of transcripts, reads were sorted by either their left most (**A, B**) or right most (**C, D**) position and plotted from top to bottom accordingly. Transcript predictions from our algorithm (**E**) and the predicted CDSs in the reference annotation file (**F**) are shown with arrows indicating the direction of transcription and with transcripts/CDSs on the different strands having different shading [light for the (+)-strand and dark for the (−)-strand].

The transcription of *LEE* operons, which are found in the E2348/69 genome, has been extensively studied. It was previously shown that for *LEE4*, a promoter upstream of *sepL* produces a *sepL-espADB* transcript that is post-transcriptionally cleaved with RNAse E to generate an *espADB* transcript and a *sepL* transcript that is then further endonucleolytically degraded (15) (Fig. 5). A putative transcriptional terminator was previously identified downstream of *espB* within *cesD2*, but it was hypothesized that there is readthrough transcription of the terminator (15). The ONT sequencing data here provided evidence for readthrough of the transcriptional terminator. Very few reads included both the *cesD2-vapB-escF* region and *sepL*, which may be an indication that processing to remove *sepL* is more efficient on the longer transcript that terminates after *espF*, although we cannot rule out that the 6 kbp transcript of the whole region was not predicted due to the size limitations of ONT direct RNA sequencing. Consistent with the latter, the 4 kbp *sepL-espADB* transcript has been detected by Northern blots in multiple studies (15, 44), yet it was very infrequently detected here. Prior 5′- and 3′-rapid amplification of cDNA ends (RACE) of *LEE4* transcripts revealed variation in transcript ends, which we also detected, with multiple reads supporting a longer transcript at the 5′-end of *sepL*, which seems to be a frequent phenomenon across all transcripts. Additionally, we predicted single CDS transcripts that encode for *espA*, *espB*, and *espF*.

**Fig 6:**
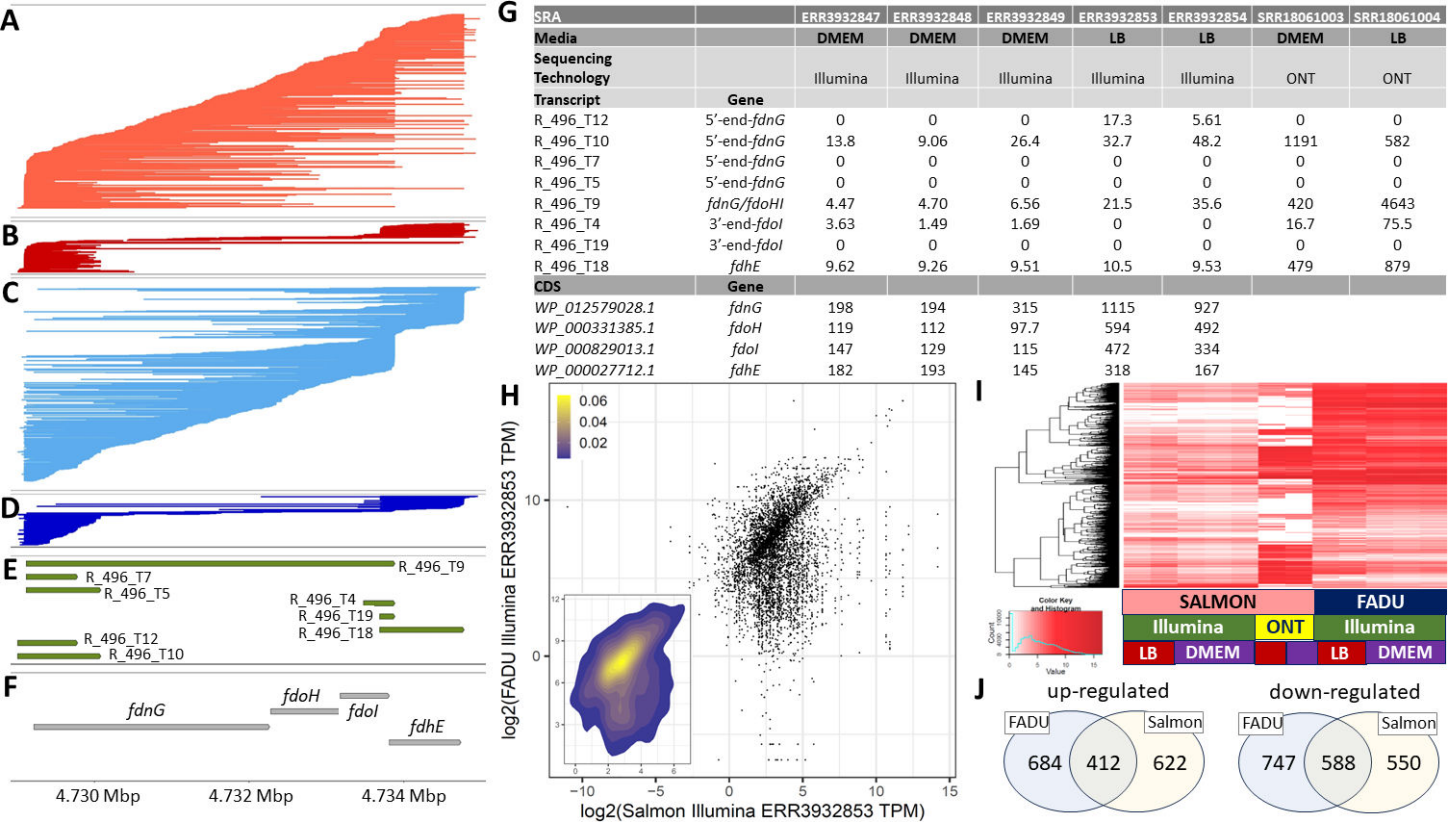
Differential expression of predicted transcripts. Reads are illustrated mapping to the plus strand of the *E. coli* E2348/69 genome (GCF_014117345.2) grown in LB (**A, C**) or DMEM (**B, D**) from 4.730 to 4.735 Mbp sorted by either their left most (**A, B**) or right most (**C, D**) position. Transcript predictions from our algorithm (**E**) and the predicted CDSs in the reference annotation file (**F**) are shown with arrows indicating the direction of transcription. Table of transcripts per million (TPM) values calculated with Salmon (31) for transcripts and FADU (18) for CDSs (**G**) for the same region shown in panels A–F. For ONT reads, only Salmon was used. Plot of the log_2_(TPM) for all CDSs and all corresponding transcripts for ERR393285 showing the discordance between TPMs calculated based on transcripts and CDSs for the same Illumina data (**H**). Heatmap clustered by genes for the log_2_(TPM) for all CDSs calculated with FADU (18) and all corresponding transcripts calculated with Salmon (31) for Illumina and ONT reads generated from LB and DMEM (**I**). Differences observed between a transcript-based differential expression analysis and a CDS-based differential expression analysis with FADU (18) are summarized showing the differences in upregulated and downregulated genes (**J**).

Using existing E2348/69 short read data from the SRA (PRJEB36845/E-MTAB-88804) and the long-read ONT data generated here, we compared differential expression results from EdgeR (49) for (i) existing CDSs predictions using FADU (18) and short reads, (ii) the transcripts predicted here using Salmon (31) and short reads, and (iii) the transcripts predicted here using Salmon (31) and long reads generated here (Fig. 6). There is discordance between the TPM (transcript per million) values calculated for all three (Fig. 6G through I) as well as assignment of genes as differential expressed in a transcript- and CDS-focused analyses of only the Illumina reads (Fig. 6J).

### Data re-use and transcripts in *Listeria monocytogenes*, *Pseudomonas aeruginosa, and Haloferax volcanii*

Through data re-use, we also predicted transcripts using published ONT data for *P. aeruginosa* strains SG17M and NN2 strains (Bacteria:gamma-Proteobacteria (37), *L. monocytogenes* strains Scott A and RO15 (Bacteria:Firmicute) (38), and *H. volcanii* (Archaea:Halobacteria) (39). All five of these strains had fewer sequencing reads than we had for *E. coli*, leading to fewer predictions of transcripts, including both mRNA and ncRNA (Table 1). Yet we were still able to predict 274–1,103 transcripts across the five strains and those transcripts were similar to the *E. coli* data with respect to mean/median/mode 3′-UTR lengths, proportion of single CDS transcripts, proportion of single transcript CDSs, size distribution of mRNA, and size distribution of ncRNA (Table 1). The 5′-UTR predictions were of similar length across the bacterial strains. However, the archaeal reads frequently did not extend beyond the 5′-end of the CDS such that monocistronic mRNAs were erroneously called ncRNAs and very long 5′-UTRS were predicted for polycistronic transcripts resulting in an increased median (Table 1). It may be that the 5′-end predictions of the CDS are flawed due to calling the longest ORF, or it may be that the *H. volcanii* UTRs are shorter than the bacterial 5′-UTRS and/or were not well captured with the ONT technology. Across all seven strains examined, two of the longest transcripts were phage transcripts and two were *nuo* transcripts (Table 1). The inclusion of *L. monocytogenes* was an important test case since it is a firmicute with leading strand transcription bias (50), which led to fewer and longer CT regions, but did not prevent high-quality transcript predictions. While there was ONT direct RNA data for further species of gamma-Proteobacteria, we limited this analysis to just two species with two strains each from this taxon. Overall, these results suggest that this simple sequencing method combined with this algorithm can be applied widely to archaeal/bacterial genomes to enable rigorous and robust transcript predictions.

### Characteristics of ONT direct RNA sequencing of *E. coli* transcripts

**Fig 7:**
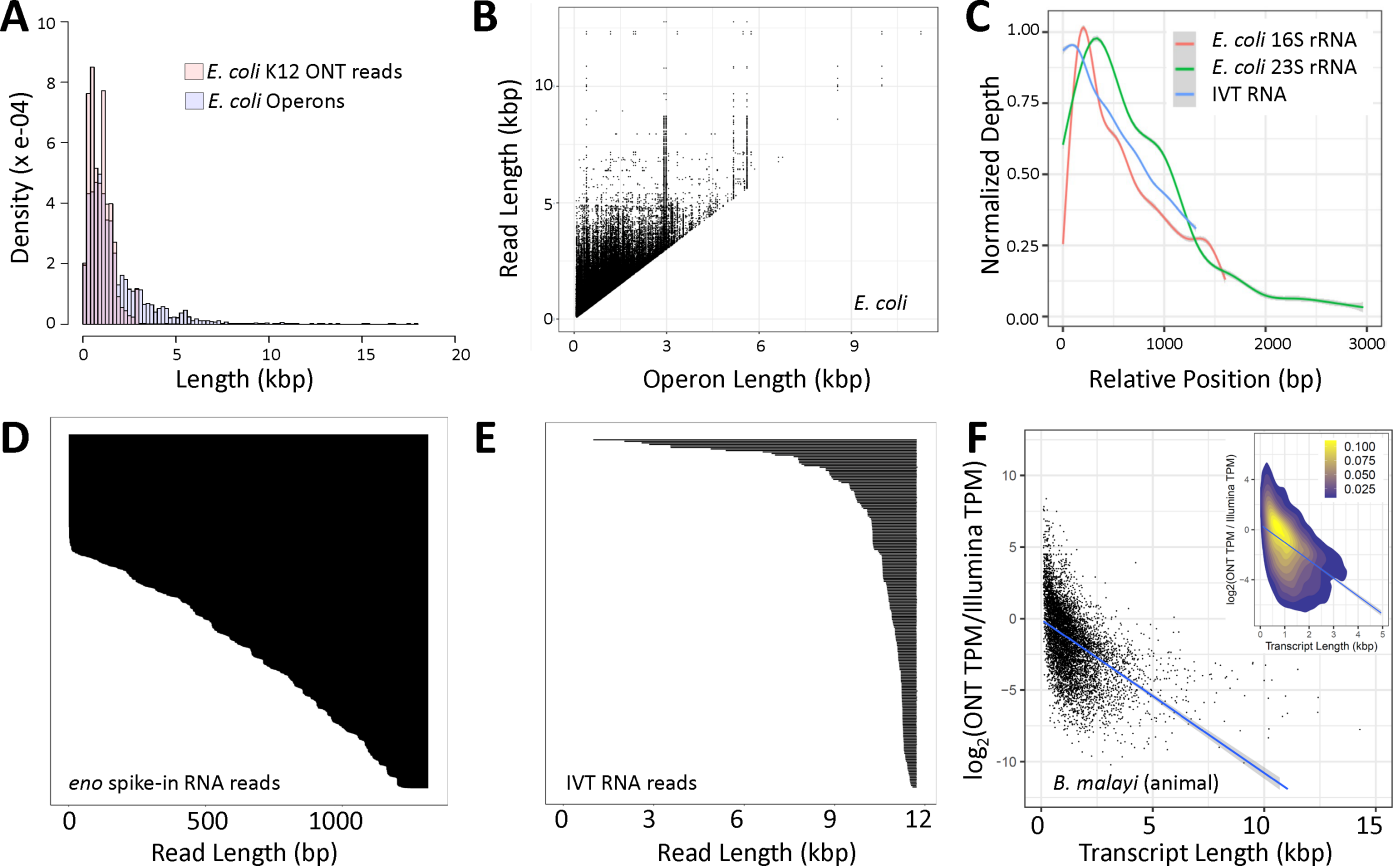
ONT sequencing characteristics that informed algorithm development. Size distribution of all of the *E. coli* K12 ONT sequencing reads aligning outside the rRNA reads compared to the distribution of predicted operons (**A**). For the 285,619 reads that are longer than the operon they map to, the length of reads is plotted relative to the size of the operon they map to (**B**). Normalized sequencing depth from the 3′-end to the 5′-end for *E. coli* K12 16S rRNA, *E. coli* K12 23S rRNA, and IVT RNA (SRR23886069), all thought to be complete, showing the 3′-bias in sequencing (**C**). Distribution of read lengths for the 1.3 kbp yeast enolase ONT spike-in (**D**) and an 11.7 kbp IVT RNA (**E**) from SRR23886069 where only reads ending at the far right position are shown. The log transformed ratios of Illumina (SRR3111494) and ONT (SRR23886071) TPM values for RNA isolated from adult female *Brugia malayi*, a filarial nematode and invertebrate animal, is compared to the transcript length, illustrating how shorter transcripts have more Illumina reads relative to ONT reads than longer transcripts (**F**). Our interpretation is that ONT sequencing is biased toward shorter transcripts. The inset uses the heat function to show the intensity of the points in the region which contains most of the data.

To develop rigorous methods and algorithms to predict these transcripts, we needed to understand the characteristics of ONT direct RNA sequencing of bacterial transcripts, which we expected to differ from sequencing of eukaryotic transcripts given the differing physical features and stability of prokaryotic and eukaryotic RNA. Overall, transcripts >5 kbp were difficult to obtain in a single read (Fig. 7A), but reads were sequenced that span most predicted operons as well as exceed the boundaries of existing operon prediction (Fig. 7A and B). While *E. coli* has known transcripts >10 kbp, we did not generate reads >9 kbp (Table 1). This could be due to laboratory handling and is, at least in part, likely due to the ONT technology since we observe that (i) this was reproducible across multiple systems and RNA molecules we know must be full length, like rRNAs (Fig. 7C), (ii) there was 5′-truncation of transcripts in 11.7 kbp full-length *in vitro* transcribed (IVT) polyadenylated RNA (Fig. 7D), and (iii) there were many incomplete reads for the 1.4 kbp yeast enolase 2 (ENO2) RNA calibration strand provided by ONT (Fig. 7E). Sequenced transcripts were also 3′-truncated (Fig. 2A through D, 4A, C and 5A through D), as previously described for ONT (28, 36, 37) and PacBio IsoSeq (30) sequencing of bacterial transcripts, possibly from (i) random fragmentation of RNA, (ii) RNA degradation, and/or (iii) incomplete transcription in a bacterial cell. Additionally, we found that shorter transcripts were preferentially sequenced relative to longer transcripts (Fig. 7F). This is despite counts/RPKMs being reported as well correlated between Illumina cDNA-based sequencing, ONT cDNA-based sequencing, and ONT direct RNA sequencing (51), as well as when nanopore direct RNA sequencing CPMs are compared to the absolute concentration of a spike-in (52).

To address incomplete reads and preferential sequencing of shorter transcripts, we first predicted transcript start/stop sites in locations where there is an overabundance of reads starting and ending. Subsequently, the actual transcripts were defined by measuring the strength of the connection between those start and stop sites using a model that supports the characteristics of truncated transcripts where smaller transcripts were preferentially sequenced. In this way, we predicted 12–15 kbp mRNAs, despite having a shorter max ONT read length (Table 1; Fig. S2).

ONT direct RNA sequencing uses changes in electrical current to detect RNA modifications including *N*6-methyladenosine (m^6^A), 5-methylcytosine (m^5^C), inosine, pseudouridine, and many more (53). At a minimum, posttranscriptional modifications were expected in bacterial tRNA and rRNA (54), but might also be present in mRNA and would lead to nonrandom changes in sequencing depth and base calling errors (55, 56). To alleviate this issue, we used a depth calculation computed assuming every base is equally present in a read using start/end positions of bed files for mapped reads. This also enables predictions in the presence of errors in the reference or sequence divergence from the reference [e.g., references (57)].

Chimeric RNA sequencing reads were detected in all samples, including chimeras between the ONT ENO2 calibration strand and sample RNA (Fig. 2H**;** Table S1). A subset of these were *in silico* chimeric reads, with a spike observed in the electrical current when analyzing the raw signal data, indicating an open pore state that was missed by the MinKNOW software (Fig. S3A and D). Others lacked this spike and could be either ligase-mediated chimeras or *in silico-*mediated chimeras where the open pore state was too short to be detected (Fig. S3B and C) (58). In our analysis, this was addressed by removing the clipped portions of mapped reads. When mapping reads to a reference genome, portions of a mapped read that do not align with the reference will be either “soft-clipped” or “hard-clipped.” A soft-clipped read has a portion that does not align to any other area of the reference (e.g., the ENO_2_ portion of an ENO_2_/mRNA chimeric read), whereas a hard-clipped read has two portions that align to different parts of the genome. For soft-clipped and hard-clipped reads, we used the primary alignment, ignoring the clipped portion of the read.

### The transcript prediction algorithm

Therefore, based on these characteristics of ONT sequencing described in the previous section, we developed tp.py, for transcript prediction written in Python. The algorithm examines each CT region separately along with the reads completely contained within that region. CT regions were initially defined through the bed input file and subsequently refined to subdivide regions based on a minimum depth cutoff (default = 2). Ultimately a region needs to have a minimum number of reads fully contained within it to be considered (default = 2). The change in depth of the sequencing reads for each genomic position of the CT region (*D*_reg_) ignoring mismatches/indels was calculated as


$$\Delta D_{reg}=D_{reg\;(n+1)}–D_{reg\;(n)}$$

Potential start and stop sites were predicted at positions where |Δ*D*_reg_| surpasses a threshold (default = 4) and always included the first and last positions of the region. ONT sequencing has issues identifying precise ends of transcripts due to polyA-trimming as well as sequencing 5′-ends, such that predicted start/stop sites in close proximity (default = 100) were grouped. Default parameters were initially established empirically upon examination of results for representative areas of the genome and confirmed to maximize sensitivity and specificity for this data set (Fig. S4).

Candidate transcripts were predicted using the Cartesian product of all predicted start and stop sites. The total read count (*N*_tot_) was calculated from the number of total reads that were mapped to all transcripts that fully contained them, allowing for mapping to multiple transcripts. The count of exclusively assigned reads (*N*_ea_) was calculated after mapping each read to the shortest transcript that fully contains it. The candidate transcripts were processed from shortest to longest computed as Ratio = *N*_ea_/*N*_tot_. If this ratio was less than the threshold (default = 0.2), the candidate transcript was discarded. If possible, reads from discarded transcripts were re-assigned to longer transcripts, and the *N*_ea_ was recalculated such that reads initially assigned to now discarded transcripts can be used to support a longer transcript. All transcripts that meet the ratio at the end of the analysis were reported in a gff annotation file and a bed file.

The algorithm runs in about an hour on a single core computer depending on the parameters and the size of the data set. We attempted to compare the results to assemblies of the ONT direct RNA reads with existing tools, including TAMA (tc_version_date_2020_12_14) (59), Cupcake (v.29.0.0) (60), and StringTie (v1.3.4d) (61), but they failed to recapitulate the complexity of the bacterial transcripts accurately (Fig. S5).

## DISCUSSION

In most bacteria, transcripts are not characterized and CDSs serve as a proxy, albeit a poor one. Here, we show that bacterial long-read transcriptome data can be used to predict bacterial transcripts using an algorithm we designed for the complexities and nuances of prokaryotic transcripts. Application of this algorithm to ONT data from four species revealed extensive transcript structural variation, transcription of RNA on both strands in some regions, overlapping transcripts, and a diversity of non-coding RNAs. The extent of transcript structural diversity highlights the need for algorithmic and analysis improvements that are important for rigorous differential expression analyses, molecular evolution analyses, and other analyses as well as laboratory experiments like making knock-outs/ins or promoter analysis. This method should enable predictions for one strain using another strain’s data, but given that we have not ascertained how much transcript structural diversity there is between strains, it may be ill-advised. For that reason, we did not, for example, use the SG17M and NN2 data to make available predictions for the research community for the frequently used *P. aeruginosa* PA01.

There were differences observed between a differential expression analysis using short/long reads as well as using transcripts/CDSs. Discordance between short and long reads may be due to: (i) shorter transcripts being preferentially sequenced relative to longer transcripts in ONT sequencing (Fig. 7F, as described below), (ii) the benefits in statistical analyses of larger numbers of Illumina reads, (iii) improper attribution of short reads to overlapping transcripts/isoforms, or (iv) differences in the incubation conditions of the cultures used in collecting the long and short read data sets. However, using only the Illumina reads, there are more differences than similarities between analyses using CDSs and those using transcripts despite using the same raw data for each analysis (Fig. 6J). This is consistent with our previous comparisons of CDS-focused and transcript-focused analyses using simulated data (19). While some of these may relate to transcripts falling just over or under an analysis threshold, others relate to transcription of an overlapping ncRNA being mis-attributed to an overlapping CDS, as seen with *fdnG* (Fig. 6A through F).

There is still room for improvement for bacterial transcript predictions, both through lab experimentation and bioinformatics. The greatest improvement in the lab would be in obtaining more full-length reads, particularly for long transcripts, which is a challenge for all long-read sequencing platforms. For ONT, the new chemistry may improve the yield and length, and further improvements to length may be possible by altering the reverse transcription method needed to remove RNA secondary structure by changing the enzyme (62). The issue of missing the last few bases of the read, which represents the 5′-end of the transcript, is a more significant issue for those looking for single base pair resolution of transcript ends. Ligating an adaptor to the read prior to sequencing shows promise in addressing that issue (52, 63). We also saw a significant amount of fragmentation at the 3′-ends that may be either incomplete transcription, 3′-degradation of transcripts, random breakage, or sequencing biases that need to be better understood. Incomplete transcription is intriguing and may reflect the fundamental biology since (i) bacterial transcription and translation are coupled and (ii) bacterial transcripts are short-lived and frequently in the process of being synthesized, since bacterial mRNAs are made at a rate of 40–80 nt/s (64), while the average mRNA half-life is only 2–10 min (65). In contrast, eukaryotic RNAs have to be spliced to create mature mRNA before being exported from the nucleus and have increased stability and a longer half-life.

When discussing taxonomy, Stephen J. Gould emphasized that “classifications both reflect and direct our thinking” (66). Going on to say that “the way we order represents the way we think” (66). Annotation has many similarities to taxonomy, and similarly genome annotation both reflects and directs our thinking. For bacteria, annotation is currently protein-centric, influencing our results and ways of thinking. Historically, this is likely due to the connection between the definition of a gene and protein, but practically it also relates to the ease with which we can computationally predict proteins. However, with new experimental methods and abilities, it is time for a sea change in bacterial genome annotation. The experimental and computational methods here are easy and quick, and thus they should be implemented widely. Additionally, there is a need for associated new ontology standards for describing transcripts and operons in annotation files that will better describe these features, similar to changes made in eukaryotic annotation files to accommodate alternative splicing and alternative transcripts (67). A harmonization of the standards for bacteria and eukaryotes would be ideal, such that there is a standard that spans the incredible biological diversity and commonalities across the domains of life.

### Conclusions

Here, we use bacterial long-read transcriptome data and a new algorithm we developed to predict transcripts from this data for two strains of three diverse bacterial species including both Gram-negative and Gram-positive bacteria. Our analysis reveals a tremendous amount of transcript structural variation, transcription of RNA on both strands in some regions, overlapping transcripts, and a diversity of non-coding RNAs, which we provide as new annotation for these genomes. Bacterial transcriptional structural variation has a richness that rivals or surpasses what is seen in eukaryotes and provides a rich new set of therapeutic and diagnostic targets.

## MATERIALS AND METHODS

### Bacterial cultures

Cryogenically preserved *E. coli* K12 MG1655 or E2348/69 were streaked onto an LB agar plate and placed in an incubator overnight at 37°C. A single colony was selected to inoculate LB broth for an overnight culture. The overnight culture was diluted 1:100 in LB broth and harvested at the optical density specified in Table S1. For DMEM, overnight cultures were grown in LB broth and diluted 1:100 in DMEM.

### RNA isolation

To isolate RNA, the Qiagen RNeasy Mini Kit was used according to Qiagen RNA Protect Reagent Handbook Protocols 4 and 7 with Appendix B on-column DNase digestion (Qiagen, Hilden, Germany). The RNA was assessed with UV-Vis spectrophotometry (Denovix DS-11, Wilmington, DE), Qubit RNA HS Assay Kit (Fisher Scientific, Waltham, MA), and TapeStation RNA Screentape (Agilent, Santa Clara, CA). RNA preparations were stored at −80°C until ready for polyadenylation and sequencing, except for the *E. coli* K12 MG1655 harvested at an optical density OD_600_ of 0.2. The RNA isolated from this one culture was treated in four different ways. For SRR27982843, 4 µg of the freshly isolated RNA was immediately polyadenylated and then taken into library preparation and sequenced, as detailed below. The leftover polyadenylated RNA was stored at −80°C alongside the original RNA isolation which had been frozen without polyadenylation. After two months, the original, unpolyadenylated RNA was thawed and polyadenylated just before library preparation and sequencing (SRR27982841). On that same day, the RNA that had been polyadenylated before being frozen was thawed and taken directly into library preparation and sequencing (SRR27982841). Four months after the original RNA isolation, the RNA that had been polyadenylated before storing at −80°C was thawed again and polyadenylated again before library preparation and sequencing (SRR27982840).

### Oxford Nanopore Sequencing

RNA was polyadenylated with *E. coli* poly(A) polymerase (M0276S, New England Biosciences, Ipswich, Massachusetts) at 37°C for 90 s to 30 min (Table S1) according to the manufacturer’s protocol and sequenced with the Direct RNA Sequencing kit (SQK-RNA002, Oxford Nanopore Sequencing, Oxford, UK) according to protocol version DRS_9080_v2_revR_14Aug2019. The prepared RNA library was loaded onto R9.4.1 flow cells (FLO-MIN106D) in a MinION device Mk1B (MIN-101B). Sequencing runs were terminated at 24 h. Fast5 files were basecalled using Guppy version 6.4.2 (68) generating FASTQ files with the high accuracy model using the rna_r9.4.1_70bps_hac config file on a GPU cluster.

### Read mapping, transcript prediction, and analysis

FASTQ files were mapped to the reference genome (Table S2) using minimap2 (v2.24-r1122; options: -ax map-ont -t 2) (69). Alignments were sorted and filtered with samtools view (v1.11; option: -F 2308) (70) generating bam files that were merged and indexed. BED files were generated with bamToBed (v2.27.1; options: -s -c 6,4 -o distinct,count) (71) and filtered with awk to remove regions with fewer than 20 reads. The tp.py algorithm was run in python (v.3.11.4). Statistics on regions, predicted transcripts, and other features were calculated with perl (v5.30.2). Perl (v5.30.2) was also used to merge the transcript and reference gff annotation files and identify mRNAs, ncRNAs, and UTRs. ONT sequencing, transcript predictions, and reference CDS predictions were visualized in R (v3.6.3). E2348/69 reads from the SRA for PRJEB36845/E-MTAB-88804 and counted against the E2348/69 with the transcript predictions presented here using Salmon (v.1.10.2) (31). Before differential expression was assessed, genes not meeting the required CPM cutoff of 5 in at least three samples were removed. The samples were grouped based on the treatment status, and differentially expressed genes were identified with EdgeR v3.30.3 using the quasi-likelihood negative binomial generalized log-linear model. Statistical significance was set at an FDR cutoff <0.05 after correction with the Benjamini-Hochberg method. A heatmap was drawn in R v4.2.1 using heatmap.3 of the *z*-score transformed log_2_(TPM) values for differentially expressed genes with the columns ordered based on a dendrogram generated using pvclust v2.2-0.

The full set of commands are described at: https://github.com/jdhotopp/tp.py-Direct-RNA-Sequencing-Manuscript-/tree/main (https://zenodo.org/doi/10.5281/zenodo.13684383).

## Data Availability

The ONT FASTQ file accessions for the data generated in this proposal are SRR18061005, SRR18061002, SRR27982845, SRR18061004, SRR18061003, SRR23886068, SRR27982844, SRR27982843, SRR27982842, SRR27982841, and SRR27982840.

## SUPPLEMENTAL MATERIAL

Figure S1 mbio.02359-24-s0001.docx DNA and RNA current in nanopore.

Figure S2 mbio.02359-24-s0002.docx *recBCD*/*ptrA* transcript predictions.

Figure S3 mbio.02359-24-s0003.docx Pore-mediated and ligase-mediated chimeras.

Figure S4 mbio.02359-24-s0004.docx Parameterization of the algorithm.

Figure S5 mbio.02359-24-s0005.docx Results from Stringtie, Tama, and Cupcake.

Table S1 mbio.02359-24-s0006.xlsx Sequencing statistics for ONT direct RNA sequencing runs with *E. coli* RNA.

Table S2 mbio.02359-24-s0007.xlsx Data used in the analysis and prediction of transcripts

ASM does not own the copyrights to Supplemental Material that may be linked to, or accessed through, an article. The authors have granted ASM a non-exclusive, world-wide license to publish the Supplemental Material files. Please contact the corresponding author directly for reuse.
