# Supplementary material for: Deciphering transcript architectural complexity in bacteria and archaea
Source: mBio. 2024 Sep 17;15(10):e02359-24. doi: 10.1128/mbio.02359-24 (PMC11481537; doi:10.1128/mbio.02359-24)

**Figure A1 – DNA and RNA current in nanopore**

*E. coli* E2348/69 ONT DNA ligation sequencing (SQK-LSK109 kit, SRR13610060) and ONT direct RNA sequencing (SQK-RNA002 kit, SRR18070404) were downloaded from the SRA. For both DNA and RNA, 1000 reads that passed the threshold were randomly selected, as well as 1000 reads with Q score < 7 for RNA to represent filtered reads. Signal levels extracted with ONT POD5 tools (<https://github.com/nanoporetech/pod5-file-format>) were plotted for each group using Python v3.8.2. P-values are based on a two-tailed *t*-test.


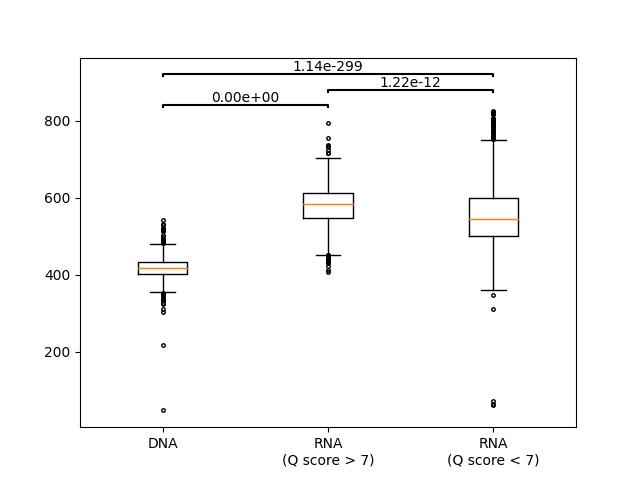

Supplement: Figure S1 — DNA and RNA current in nanopore. [file mbio.02359-24-s0001.docx]
