# Supplementary material for: Deciphering transcript architectural complexity in bacteria and archaea
Source: mBio. 2024 Sep 17;15(10):e02359-24. doi: 10.1128/mbio.02359-24 (PMC11481537; doi:10.1128/mbio.02359-24)
Supplement: Figure S2 — recBCD/ptrA transcript predictions. [file mbio.02359-24-s0002.docx]

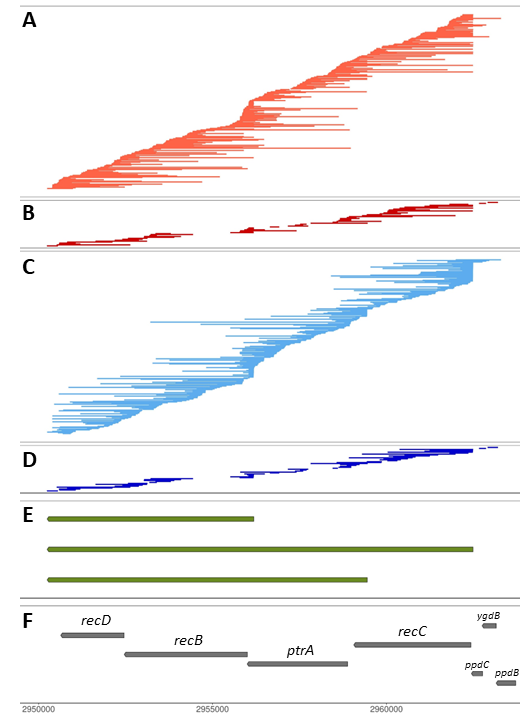


**Figure A2 – *recBCD/ptrA* Transcript Predictions**

Minus-strand ONT direct RNA sequencing reads (shown as lines) are mapped from ~2.950-2.965 Mbp in the *E. coli* K12 genome (NC_000913.3), which corresponds to a region encoding RecBCD and PtrA. Reads are sorted by their transcription stop site for *E. coli* K12 grown in rich LB media (left sorted, **A**; right sorted, **C**) and DMEM media (left sorted, **B**; right sorted, **D**). Our algorithm predicts 3 transcripts (**E**), and 7 CDSs in the reference NC_000913.3 annotation file are illustrated (**F**). While there are no ONT reads that span the entire *recBCD/ptrA* region, there is sufficient evidence to call this transcript. This is because, after removing reads wholly contained within a predicted *recBD* transcript and a *ptrA/recBD* transcript there were sufficient reads remaining to predict a transcript that spans *recBCD/ptrA*.
