# Supplementary material for: Deciphering transcript architectural complexity in bacteria and archaea
Source: mBio. 2024 Sep 17;15(10):e02359-24. doi: 10.1128/mbio.02359-24 (PMC11481537; doi:10.1128/mbio.02359-24)
Supplement: Figure S3 — Pore-mediated and ligase-mediated chimeras. [file mbio.02359-24-s0003.docx]

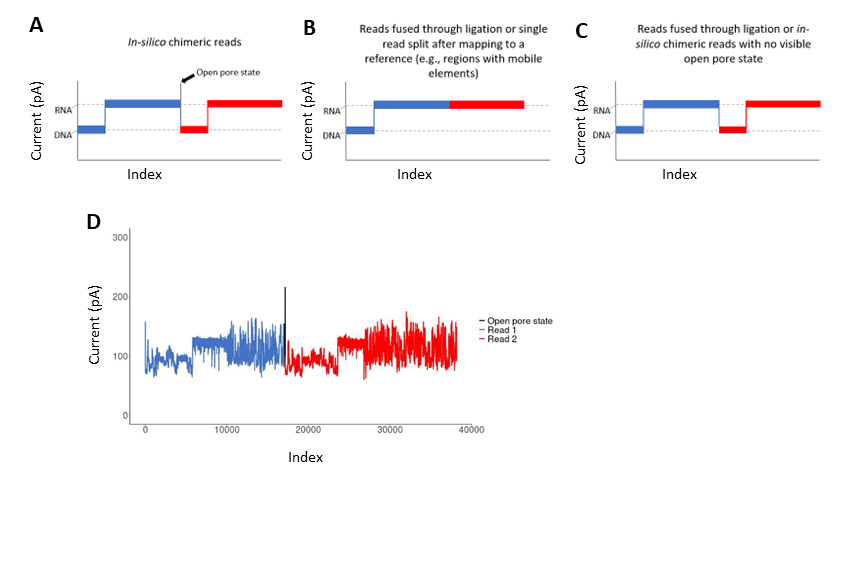
**Figure A3 – Pore-mediated and ligase-mediated chimeras**

Chimeric sequencing reads observed in ONT direct RNA sequencing data can theoretically be generated by (A) two reads entering the pore in tandem with an open pore state that is not detected, (B) two RNA molecules being fused through ligation in vivo, in vitro during library construction, or a mapping artifact when there is a rearrangement in the genome reference, and (C) two fragments fused by ligation following adapter ligation during library construction. (D) A chimeric read from a sequencing run that shows that an open pore state (black) was missed between the first read (blue) and the second read (red). For both reads, the characteristic DNA adaptor with a lower current is observed followed by a higher plateau that is the polyA tail being sequenced. The open pore state is a spike from increased current when the pore is open between RNA molecules sequenced.
