# Supplementary material for: Deciphering transcript architectural complexity in bacteria and archaea
Source: mBio. 2024 Sep 17;15(10):e02359-24. doi: 10.1128/mbio.02359-24 (PMC11481537; doi:10.1128/mbio.02359-24)
Supplement: Figure S4 — Parameterization of the algorithm. [file mbio.02359-24-s0004.docx]

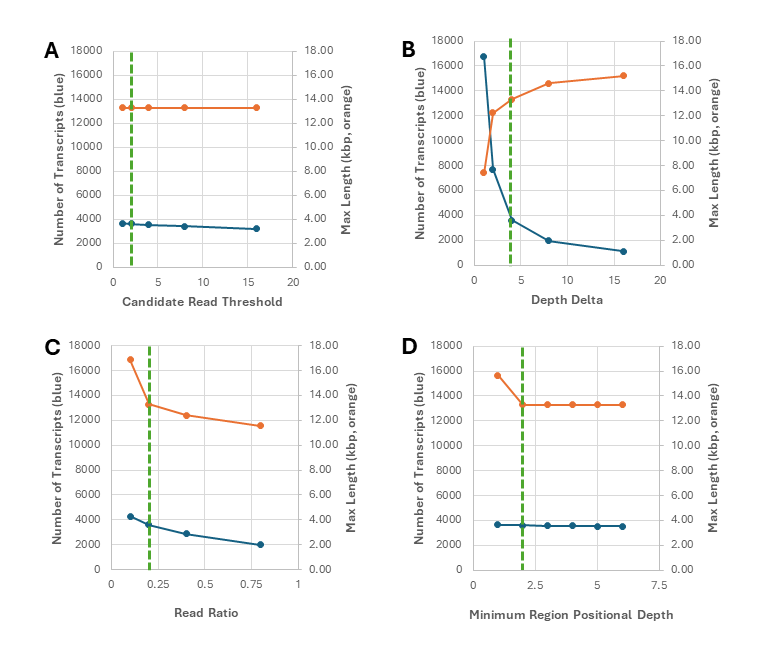


**Figure A4—Parameterization of the algorithm**

Default parameters were initially determined empirically and then verified to maximize specificity and sensitivity using all *E. coli* data by comparing the number of transcripts predicted (blue, left axis) and the length of the longest transcript (orange, right axis) while varying a single parameter and holding all other values at the default. Default values are marked with a dashed green line in the plots. It is likely that the best parameters will vary by the sequencing depth, nuances in the sequencing technology, and organismal characteristics that include the coding density and coding strand bias. The minimum depth at all values tested gave the same values as default, interpreted to be due to intentional redundancies in the parameters that facilitate customization of the analysis, in this case the minimum region positional depth.
