# Supplementary material for: Deciphering transcript architectural complexity in bacteria and archaea
Source: mBio. 2024 Sep 17;15(10):e02359-24. doi: 10.1128/mbio.02359-24 (PMC11481537; doi:10.1128/mbio.02359-24)
Supplement: Figure S5 — Results from Stringtie, Tama, and Cupcake. [file mbio.02359-24-s0005.docx]

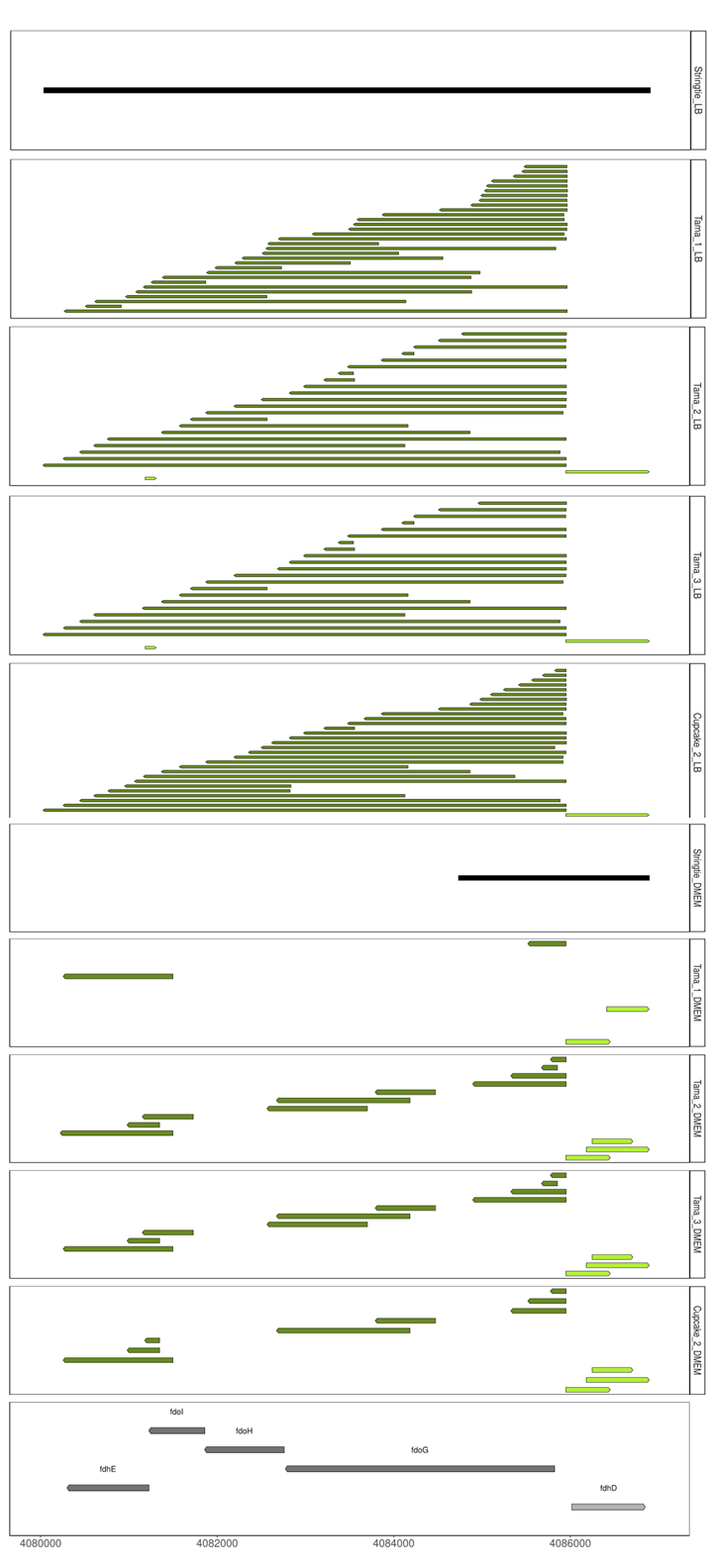


**Figure A5 – Results from Stringtie, Tama, and Cupcake**

Transcript predictions resulting from Stringtie, Tama, and Cupcake are shown for *E. coli* K12 for the same region as presented in Figure 4. Plots are labeled on the right side according to the notation in the github page that fully describes how they were run with those ending in LB resulting from only the K12 LB data and those ending in DMEM resulting from only the K12 DMEM data. Stringtie is splicing focused, and since bacteria do not have splicing it is unsurprising that it could not predict transcript structures, largely yielding transcript predictions corresponding to zero-depth regions across the genome. Whether default (Tama 1) or user-defined parameters were used (Tama 2 and 3), Tama frequently over-called transcripts, particularly in regions with higher sequencing depth. With default parameters (Cupcake 1), Cupcake tends to under-call transcripts because ONT reads get filtered out of analysis due to higher degree of mismatches, and in this region no results were reported. When the parameters were adjusted to better fit ONT reads (Cupcake 2), Cupcake produced results similar to TAMA.
